# Effects of Hyperoxia and Hyperoxic Oscillations on the Proteome of Murine Lung Microvascular Endothelium

**DOI:** 10.3390/antiox11122349

**Published:** 2022-11-28

**Authors:** Akos Tiboldi, Eva Hunyadi-Gulyas, Peter Wohlrab, Johannes A. Schmid, Klaus Markstaller, Klaus Ulrich Klein, Verena Tretter

**Affiliations:** 1 Department of Anesthesia, General Intensive Care and Pain Management, Medical University of Vienna, 1090 Vienna, Austria; 2 Laboratory of Proteomics Research, Biological Research Centre, Eötvös Loránd Research Network (ELKH), Temesvári krt. 62, H-6726 Szeged, Hungary; 3 Department of Vascular Biology and Thrombosis Research, Center for Physiology and Pharmacology, Medical University Vienna, Schwarzspanierstraße 17, 1090 Vienna, Austria

**Keywords:** acute respiratory distress syndrome, hyperoxic acute lung injury, lung microvascular endothelial cells, ventilator-induced lung injury, hyperoxia, oxygen oscillations, proteomics, enrichment analysis

## Abstract

Patients presenting with insufficient tissue oxygenation and impaired lung function as in acute respiratory distress syndrome (ARDS) frequently require mechanical ventilation with supplemental oxygen. Despite the lung being used to experiencing the highest partial pressure of oxygen during healthy breathing, the organ is susceptible to oxygen-induced injury at supraphysiological concentrations. Hyperoxia-induced lung injury (HALI) has been regarded as a second hit to pre-existing lung injury and ventilator-induced lung injury (VILI) attributed to oxidative stress. The injured lung has a tendency to form atelectasis, a cyclic collapse and reopening of alveoli. The affected lung areas experience oxygen conditions that oscillate between hyperoxia and hypoxia rather than remaining in a constant hyperoxic state. Mechanisms of HALI have been investigated in many animal models previously. These studies provided insights into the effects of hyperoxia on the whole organism. However, cell type-specific responses have not been dissected in detail, but are necessary for a complete mechanistic understanding of ongoing pathological processes. In our study, we investigated the effects of constant and intermittent hyperoxia on the lung endothelium from a mouse by an in vitro proteomic approach. We demonstrate that these oxygen conditions have characteristic effects on the pulmonary endothelial proteome that underlie the physiological (patho)mechanisms.

## 1. Introduction

Acute respiratory distress syndrome (ARDS) is a life-threatening condition caused either by extra-pulmonary or intrapulmonary events such as sepsis, aspiration, pneumonia or trauma. It is characterized by activation of the immune system and severe inflammation of the lungs, increased alveolar capillary permeability leading to pulmonary edema, dysfunctional blood clotting and loss of ventilated lung area. Patients present with impaired gas exchange, hypoxemia, decreased carbon dioxide (CO_2_) elimination and reduced lung compliance [1,2,3]. A standard treatment of ARDS is invasive mechanical ventilation accompanied with supplemental oxygen to ensure sufficient tissue oxygenation. Ventilation and high fractions of inspired oxygen (FiO_2_) by themself bear the risk of further damaging the lung, known as ventilator-induced lung injury (VILI) and hyperoxic acute lung injury (HALI) [4]. VILI is caused by lung overdistention resulting in “volutrauma”, and by shear stress due to cyclic collapse and the reopening of diseased lung areas with a low functional residual capacity (“atelectotrauma”). Physical demand together with the initial disease result in inflammatory states (“biotrauma”), which are probably a prime cause of death in these patients [5]. An additional threat are secondary pulmonary infections that are facilitated by invasive ventilation. Oxygen therapy aims to prevent states of hypoxemia, but since the 1960s it has been increasingly recognized that oxygen in high concentrations (FiO_2_ > 0.7) is harmful to the lung tissue. Patients with severe ARDS often require hyperoxic therapy and are therefore at risk of developing HALI. Underlying pathological mechanisms and triggers of tissue damage due to HALI have been characterized to some extent in animal models [6]. However, clinical conditions might be frequently divergent [7]. Cyclic recruitment and de-recruitment of atelectasis have been shown to result in oscillations of the arterial partial pressure of oxygen, which can be transmitted to remote organ systems via the vascular system [8]. Such oxygen oscillations (in the range between severe states of hypoxia and hyperoxia) most likely trigger different molecular responses, posing the risk of an independent and hitherto uncharacterized (patho) mechanism. A primary recipient of these oxygen conditions, and at the same time a primary transmitting organ for downstream effects, is the pulmonary endothelium.

In our study, we aimed to investigate specific oxygen condition-dependent cell responses in murine lung endothelial cells with a proteomic and bioinformatic approach. We used murine primary cell cultures that were directly exposed to these oxygen conditions in order to eliminate other influencing factors, such as shear stress or primary inflammation.

## 2. Methods

### 2.1. Isolation of Mouse Lung Endothelial Cells and Cell Culture

Mouse primary lung endothelial cells were isolated after digesting lung tissue from adult C57B/L6 mice (aged 6–8 weeks) with collagenase II by magnetic separation using bead-coated antibodies for endothelial cell surface markers CD31 and ICAM1, according to the method described in [9]. six-well cell culture dishes were precoated with 2% gelatin and 10 μg/mL fibronectin, and cells were plated in M199 medium (ThermoFisher, Waltham, MA, USA), 20% fetal calf serum superior (Biochrom GmbH, Berlin, Germany), 30 μg/mL endothelial cell growth supplement from bovine pituitary (Sigma-Aldrich, Burlington, MA, USA), 5 U/mL heparin, and antibiotics. Cells were expanded for 2 weeks in vitro and repurified by magnetic separation prior to seeding for gas exposure. Three individual cell cultures (prepared from 30 mice each) were used for label-free proteomic analysis. Another three independent cell cultures (prepared from 15 mice each) were used for mRNA expression analysis by qRT-PCR.

### 2.2. Cell Exposure to Different Oxygen Conditions

Trypsinized cells were plated into 6-well plates containing gas-permeable membranes (imaging plates; Zellkontakt, Nörten-Hardenberg, Germany) and transferred to custom-made boxes 24 h after plating (as described in [10]). Cells were exposed to different O_2_-conditions supplied by premixed gas bottles: (1) 21% O_2_—5% CO_2_—74% N_2_; (2) 95% O_2_, 5% CO_2_; (3) 0–95% O_2_ oscillations—5% CO_2_—rest N_2_, with a frequency of 6 oscillations per hour (as described in [11]).

### 2.3. Sample Preparation for Proteomic Analysis

After 24 h and 72 h of exposure to different oxygen conditions, cells were lysed with RIPA buffer (25 mM Tris/HCl pH 7.4; 150 mM NaCl; 0.5% sodium deoxycholate; 0.1% SDS; 1% NP-40; PIERCE protein inhibitor tablet). Proteins were quantified by a bicinchoninic acid-based protein assay (PIERCE- ThermoFisher, Waltham, MA, USA) and precipitated according to a modified method of Wessel and Fluegge [12]. 200 μg of protein was redissolved in 50 μL of 0.5% RapiGest (Waters Corporation, Milford, MA, USA) in 50 mM ammonium bicarbonate pH 7.8 and used for analysis.

### 2.4. Protein Identification and Semi-Quantitative Comparison by 2D-Liquid Chromatography/Mass Spectrometry (2DLC-MS)

We have described our proteomic methodology in a previous paper from our lab [13]. Individual steps and settings are summarized here.

### 2.5. Enzymatic Digestion

Proteomic investigations were performed in three biological replicates. Protein samples were digested in-solution after the reduction in disulfide bridges and alkylation (reduction: 2 μL 100 mM DTT; 56 °C for 30 min; alkylation: 4 μL of 100 mM iodoacetamide; 40 min room temperature in the dark; quenching: 0.2 μL 100 mM DTT). Digestion was performed with 2.4 μL of 1 μg/μL trypsin at 37 °C overnight, and terminated by adding 12 μL of 10% formic acid.

### 2.6. 2D LC-MSMS Analysis

High pH reversed phase (RP) fractionation was performed using an Eldex micro HPLC pump (Sunchrom, Friedrichsdorf, Germany) on a RP column (Phenomenex, Kinetex 5u EVO C18 100A, 2.1 × 100 mm; Cat# 00D-4622-AN). Mobile phases were: A: 10 mM (NH_4_)HCO_3_, pH = 10; B:10% A in acetonitrile. Elution was performed at a flow rate of 150 μL/min with the following gradient: 5–40% B in 10 min, 40–95% B in 2 min, 95% B for 3 min 95–5% B in 2 min and 5% B for 7 min. 48 fractions were collected from 1 to 25 min and 4-4 fractions were combined (1, 13, 24, 37; 2, 14, 26, 38, etc.). This finally resulted in 12 fractions. Each of them was dried in a vacuum centrifuge, resolved in 0.1% formic acid in water and subjected to nanoLC-MSMS analysis on an LTQ-Orbitrap Elite (Thermo Fisher Scientific, Waltham, MA, USA) mass spectrometer. NanoUPLC runs were performed on a Waters nanoAcquity ULC system (Waters, Milford, MA, USA), using gradient elution after trapping the samples onto the trap column (186007238 Waters Symmetry C18, 0.180 mm × 20 mm, 5 μm, 100 Å) with 3% of B (mobile phase A: 0.1% formic acid in water; mobile phase B: 0.1% formic acid in acetonitrile) for 2 min with 10 μL/min flow rate. The analytical separation was performed with the following gradient elution: 3–10% B in 5 min and to 40% in 32 min, followed by double wash at the end of the gradient in order to reduce any carry over for the next run. The flow rate was 200 nl/min, the column (186003545 Waters BEH130 C18, 0.075 mm × 250 mm, 1.7 μm, 130 Å) was kept at 60 °C.

We applied data-dependent analyses: the 20 most intense peaks were selected for ion-trap CID after each survey scan. The survey spectra were measured in the Orbitrap (mass range: 380–1400 *m*/*z*; resolution: 120,000 @ 400 *m*/*z*), while the CID MS2 spectra were detected in the ion trap. Selected precursor masses were dynamically excluded for 15 s to facilitate more comprehensive analysis of the samples.

### 2.7. Data Evaluation

We used Proteome Discoverer (version 1.3, Thermo Fisher Scientific, Waltham, MA, USA) to generate msms peaklist files and our in-cloud ProteinProspector (version 5.18.0, UCSF, San Francisco, CA, USA) database search engine for protein identification. Peaklist files related to one sample were merged and used for searches against the bovine and mouse proteins from UniProtKB.2015.4.16. database, supplemented with their random sequences (109,150 sequences). Only fully specific tryptic peptides with a maximum of one missed cleavage site were considered. Carbamidomethyl cysteine as constant, methionine oxidation, peptide N-terminal pyro-glutamine formation from glutamine, and protein N-terminal acetylation were set as variable modifications. Error tolerance for precursor ions was set to 10 ppm and 0.6 Da for fragment masses. Maximum 2 modifications per peptides were allowed.

### 2.8. Semi-Quantitative Evaluation by Spectral Counting

Because there are a lot of proteins with a high degree of homology between mouse and bovine (source of bovine proteins: fetal calf serum of cell culture medium), it is challenging to decide the origin of the proteins. Database search results were acquired without homology and set MOUSE as the preferred species. The calculated false discovery rates (FDR) were less than 1%. In at least one of the groups, the protein from all of the three replicates had to be identified and at least 5 unique peptides had to match the protein in 2 cases from the 3 replicates. The semi-quantitative analysis relied on the number of spectra identifying the particular protein (peptide count/total number of peptide spectrum match) of the sample. To avoid the calculation problem when a protein showed spectral count zero in one of the samples, the following formula was used to calculate the relative spectral counts: RPC = (*n* + *f*)/(*t* + *f*), where RPC is the relative peptide count, *n* is the number of spectra identifying the protein, *t* is the total number of identified spectra in the sample, *f* is a correction factor, set to 1.

### 2.9. Functional Protein-Interaction and Pathway Enrichment Analysis

The lists of proteins that were identified as significantly changed in expression after treatment with constant or intermittent hyperoxia were uploaded, including fold change values, to the web-based analysis platform NetworkAnalyst (3.0) [14] (www.networkanalyst.ca (accessed on 7 April 2022) using MOUSE as species and ID (Uniprot) as an identifier. Using this platform, differentially expressed proteins were compared with the protein interactome of the STRING database (cut-off: 700) generating first- and higher-order networks with the differentially regulated proteins from the list as seeds. The networks were downloaded as graphml file and imported into Cytoscape 3.8.2 (The Cytoscape Consortium, San Diego, CA, USA) [15]. The *stringApp* of Cytoscape was used to “STRINGify” the network for the species *mus musculus* followed by functional enrichment of pathways and gene ontologies. The STRING enrichment table was ranked according to FDR values and filtered for Reactome, KEGG pathways and Gene Ontologies. Detailed results of the individual analysis are provided as Supplemental Information (Supplementary Data S2).

### 2.10. Quantitative Real-Time PCR

For quantification of protein gene expression, mRNA was isolated from cells after exposure to different oxygen conditions using the Rneasy plus kit (Qiagen, Hilden, Germany). Reverse transcription was performed using qScript Supermix (Quanta Biosciences, Gaithersburg, MD, USA), and resulting cDNA was analyzed by quantitative real-time PCR on a RotorGene Q (Qiagen, Hilden, Germany). Changes in gene expression were calculated relative to the control condition (21% O_2_) using the ΔΔCt method and *Actb* or *Gadph* as house-keeping gene. Primer sequences are provided in Supplementary Data S1.

### 2.11. Statistical Analysis

Proteomic data were generated from three independent cell preparations. Fold changes in protein levels were statistically evaluated by Student’s *t*-test (two sample *t*-test, assuming unequal variances) to decide whether the difference between the two groups (with 3 replicates) was significant (*p* < 0.05). Comparing different groups meant levels of individual proteins under constant or intermittent hyperoxia relative to protein levels after culture under normoxia (21% O_2_) for the same period of time (24 h or 72 h).

Since there was a significant variance between groups, the averages of the relative peptide counts were compared and at least two-fold changes were considered as real differences. As it was assumed that most of the proteins did not show significant changes, the relative peptide count ratio normalization by the median value was performed.

qPCR analysis was performed from three independent experiments. The graphs show the results from one representative experiment analyzed from 3 individual wells (triplicates) of gas-exposed cell culture dishes. qPCR data were statistically evaluated by one-way ANOVA followed by Dunnett’s multiple comparison test using GraphPad Prism 8.0 software (GraphPad Software, San Diego, CA, USA). Mean expression levels were further compared to the value 1.0 by using a one-sample *t*-test. In all statistical tests *p* < 0.05 was regarded as the threshold for significant results.

## 3. Results

In order to analyze robust changes in protein expression in response to different oxygen conditions, we exposed cells initially for 24 h and as a follow up for a total of 72 h. Protein expression levels under constant hyperoxia (95% O_2_) and oxygen oscillations (0–95% O_2_) were directly compared to expression levels in cells that were exposed to normoxia (21% O_2_) for the same period of time (fold-change of expression).

### 3.1. Changes in Protein Expression over Prolonged Periods of Severe Constant Hyperoxia

24 h of exposure to constant severe hyperoxia significantly changed the expression of 59 proteins (=Differentially Expressed Proteins, DEPs), of which 49 were upregulated and 10 were downregulated (see Table 1A). 

Differentially expressed proteins were analyzed on the web-based platform NetworkAnalyst 3.0 [14]. To that end, DEPs were compared with the protein interactome of the STRING database (established by a consortium of the Swiss Institute of Bioinformatics, Novo Nordisk Foundation Center Protein Research and European Molecular Biology Laboratory, EMBL). The STRING database comprises known and predicted protein-protein interactions, which include physical and functional associations derived from genomic context predictions, high-throughput lab experiments, coexpression data, and automated text mining. Data included in the STRING database stem from other primary databases, from computational predictions and knowledge transfer between organisms. A first order network was computed that comprises the DEPs (as seed proteins) and the predicted STRING interactome as described in the Methods section. This network was downloaded as graphml-file, imported into Cytoscape 3.9.1. software, and “STRINGified” with the *stringApp*, followed by a functional enrichment analysis.

**Figure 1:**
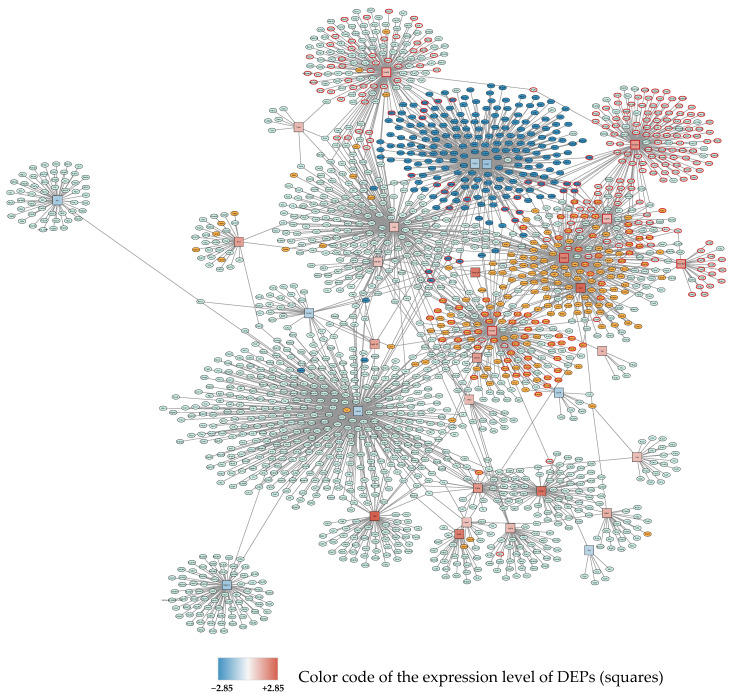
Subnetwork 1 of DEPs and their protein interaction partners after 24 h exposure to 95% O_2_. Differentially expressed proteins (seed proteins of the network) are shown as squares, with the fill color corresponding to the expression level (blue: downregulated; red: upregulated; the intensity of the color represents the degree of down- or upregulation as indicated in the legend). Interacting proteins not belonging to the seed protein list are shown as ellipses. Primary enriched pathways and functions with FDR < 0.05 are highlighted: Upregulated: cell cycle (orange), metabolism of RNA and RNA splicing (red borders), Downregulated: ribosomes (dark blue).

Enriched KEGG and Reactome pathways, as well as gene ontologies for biological functions, were visualized in color-coded networks. The largest subnetwork showing nodes and first neighbors is depicted in Figure 1. 

**Figure 2:**
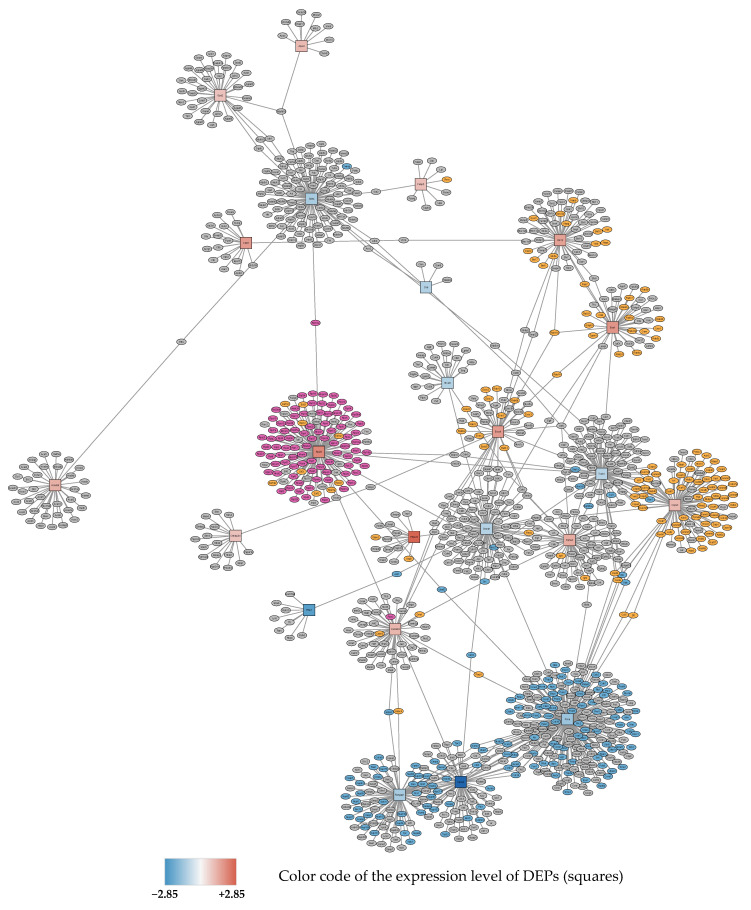
Subnetwork 1 of functionally associated DEPs and their interaction partners after 72 h exposure to 95% O_2_. Upregulated proteins are shown as red squares, downregulated nodes are shown as blue squares as described in Figure 1. Primary enriched pathways with FDR < 0.05 are highlighted: upregulated: metabolism of proteins (orange), ribosome (purple), downregulated: cell cycle (blue).

At 72 h 95% O_2_, 15 proteins were significantly downregulated and 21 proteins upregulated (see Table 1B). Deferred protein-protein interaction analysis gave 4 subnetworks. The largest subnetwork showing nodes and first neighbors is depicted in Figure 2.

### 3.2. Bioinformatic Analysis: Pathways and Enrichment Analysis

Functional enrichment analysis was performed in order to identify processes and pathways, that are overrepresented compared to background. We used Gene Ontology (GO), KEGG, and Reactome database, and performed enrichment analysis using the STRING-App of the Cytoscape software. Proteins entered in the query were those which were significantly changed in expression under different oxygen conditions at the time points 24 h and 72 h. A complete list of statistically enriched pathways is found in the Supplemental Information (Supplementary Data S2).

Major enriched biological processes and pathways associated with *upregulated* proteins after 24 h 95% O_2_ were RNA metabolism, cell cycle, mRNA splicing, cellular stress response, tight junctions and focal adhesions, neddylation, interleukin signaling, and apoptosis.

Biological processes associated with *downregulated* proteins at 24 h 95% O_2_ included translation, Rho and Ras (small GTPases) protein signal transduction, the citric acid (TCA) cycle and respiratory electron transport/oxidative phosphorylation, regulation of the actin cytoskeleton, VEGFA-VEGFR2 pathway, platelet activation, and cell junction organization.

72 h 95% O_2_ revealed enriched the biological processes of *upregulated* proteins primarily related to the metabolism of proteins, ribosomes and cellular transport mechanisms. Predominant downregulated processes after 72 h 95% O_2_ were cell cycle, DNA repair, TCA cycle and respiratory electron transport, and cellular stress response.

### 3.3. Changes in Protein Expression over Prolonged Periods of Hypoxic/Hyperoxic Oscillations

Detected changes of protein expression after 24 h of 0–95% O_2_ oscillations resulted in significantly fewer DEPs when compared to 24 h 21% O_2_. A total of 12 proteins were downregulated and 3 proteins were upregulated (see Table 2A).

**Figure 3:**
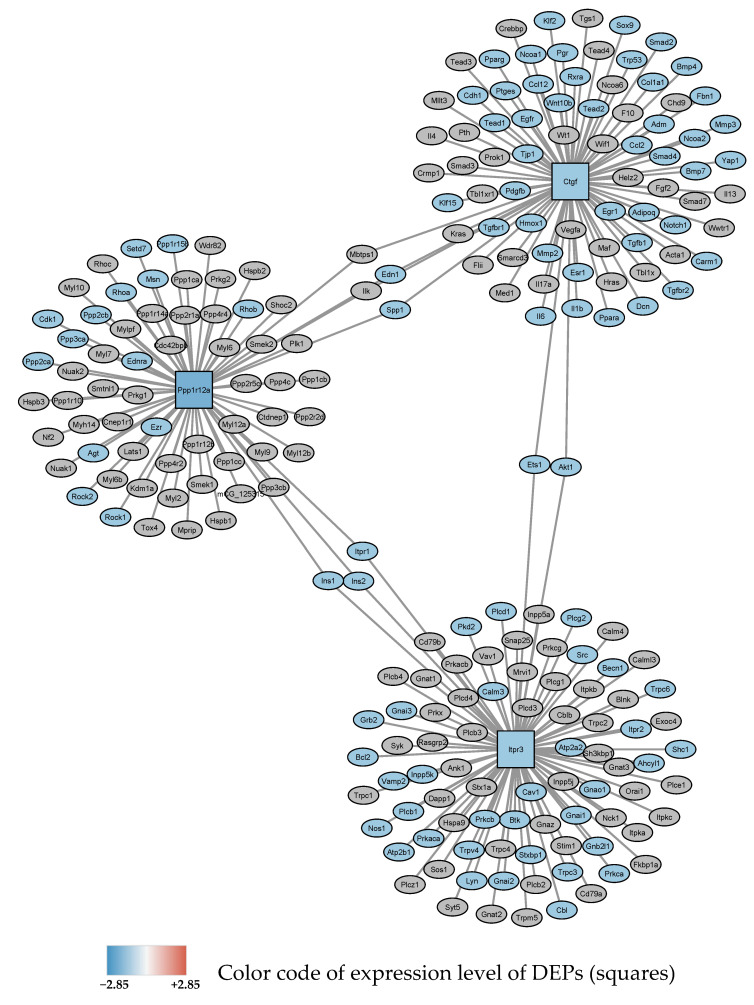
Subnetwork 1 of functionally associated DEPs and their interaction partners after 24 h exposure to 0–95% O_2_ oscillations. Blue square nodes are seed proteins found to be downregulated. The most significant enriched pathway associated with the interacting proteins with FDR < 0.05 is shown as blue ellipses and represents: “response to oxygen-containing compound”.

Protein interaction analysis resulted in 6 small subnetworks The first subnetwork showing nodes and first neighbors is depicted in Figure 3.

**Figure 4:**
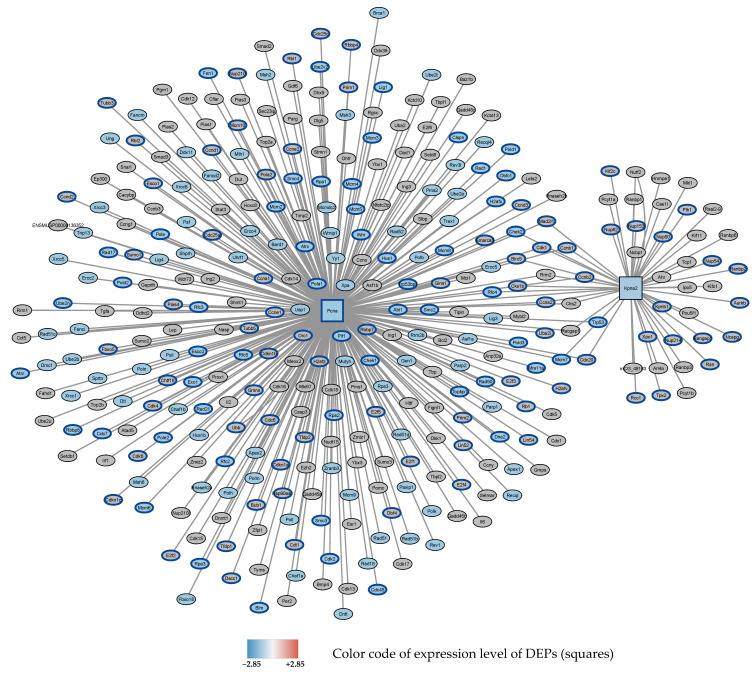
Subnetwork 1 of functionally associated DEPs and their interaction partners after 72 h exposure to 0–95% O_2_. Squares: seed proteins; ellipses: interaction partners. Colors of the ellipses highlight the following enriched pathways with FDR < 0.05: Downregulated: DNA repair (blue fill color) (GO database), cell cycle (blue border) (Reactome data base).

After 72 h, 0–95% O_2_ oscillations, 4 proteins were found to be downregulated and 5 proteins were upregulated (see Table 2B). DEPs showed functional relationship in 4 subnetworks. Subnetwork 1 showing nodes and first neighbors is depicted in Figure 4.

### 3.4. Bioinformatic Analysis: Pathways and Enrichment Analysis

Oxygen oscillations (0–95% O_2_) at 24 h revealed a downregulation of cellular response to oxygen-containing compounds, cellular calcium homeostasis, the regulation of phosphorylation, and platelet activation.

After 72 h 0–95% O_2_ oscillations, upregulated proteins were related to neddylation, post-translational protein modification, and endocytosis, while downregulated proteins were related to DNA repair, cell cycle, and p53 signaling pathway.

### 3.5. Changes in Protein Expression over Prolonged Periods of Cultivation under Normoxic Conditions

Primary mouse lung endothelial cells in culture tend to dedifferentiate quite rapidly assuming a more mesenchymal phenotype over longer culture periods (Endothelial-mesenchymal transition, Endo-MT). This process will also affect changes in protein expression. We therefore also compared protein expression at time points 24 h and 72 h to baseline values (at time = 0 h), when cultured under normoxic conditions (21% O_2_). At 24 h, only 5 proteins were differently expressed: 1 downregulated protein (Caldesmon-Cald 1), and 4 upregulated proteins (Plasminogen Activator Inhibitor, PAI-1, Transforming Growth Factor TGF-ß1ITP, Cadherin Cadh5, Glutamine–fructose-6-phosphate transaminase (isomerizing) GFAT-1) (Table 3A). These genes are involved in vascular development, angiogenesis, tissue remodeling, growth, cell migration, developmental maturation, tube development, cell fate commitment, regulation of lymphocyte activation, epithelial to mesenchymal transition, endothelial cell proliferation and migration, cell–cell junction organization, cell-matrix adhesion, MAPK pathways, protein phosphorylation, and proteoglycan biosynthesis.

At 72 h cultivation at 21% O_2_, more proteins were changed in expression (19 upregulated, 1 downregulated) (Table 3B). Functional protein interaction analysis gave a network with 16 seeds (data not shown). Enriched cellular processes include intracellular signal transduction, cytoskeletal organization, cell migration, MAPK cascade, cell-substrate adhesion, actin filament-based processes, cell adhesion, angiogenesis, cell proliferation, cell projection assembly, and Rho signal transduction. These are biological processes, which are expected in the context of Endo-MT.

In order to identify oxygen-dependent changes in protein expression and to exclude culture-dependent changes, we referenced all protein expression changes relative to 21% O_2_ at the same time point. Results from enrichment analysis show that this procedure eliminated influences from the dedifferentiation of primary cells.

### 3.6. Time-Dependent Expression of mRNA Levels of Selected Proteins as Quantified by qRT-PCR

The Eepression of mRNA levels of selected DEPs involved in key cellular processes, as described in Table 4, was analyzed by qRT-PCR at time points 4 h, 24 h and 72 h.

**Figure 5:**
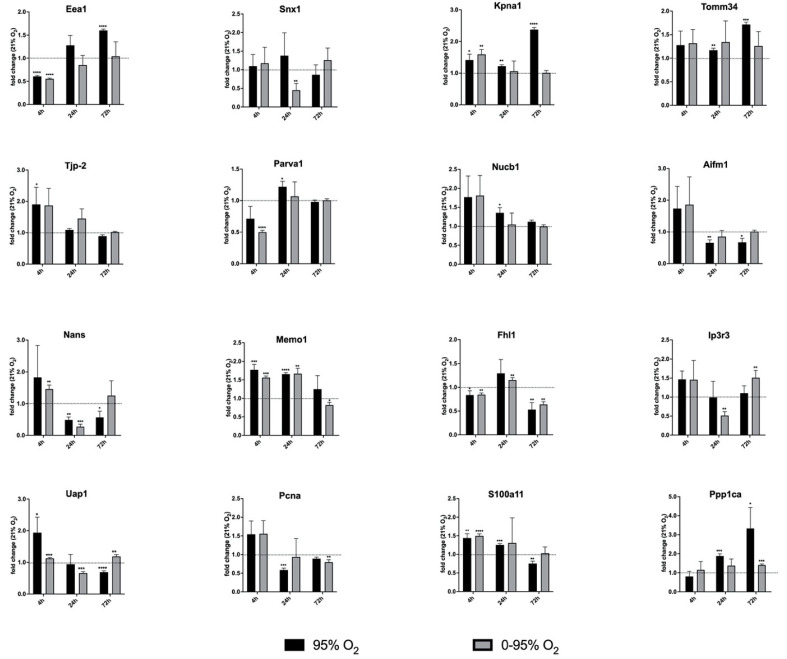
Expression of mRNA levels of selected DEPs as analyzed by qRT-PCR. The mRNAs of the following DEPs as identified from proteomic analysis were quantified: Eea1 (Early Endosomal Antigen-1), Snx1 (Sorting Nexin-1), Kpna1 (importin-α1), Tomm34 (Translocase of Outer Mitochondrial Membrane 34), Zo-2, Tjp2 (Zona Occludens-2, Tight junction protein-2), Parva1 (Parvin-α1), Nucb1 (Nucleobindin-1), Aifm1 (Apoptosis Inducing Factor-1, mitochondrion-associated), Nans (N-Acetylneuraminate Synthase), Memo1, Fhl1 (Four and a half LIM domain protein 1), Ip3r3 (Inositol-1,4,5-triphosphate Receptor 3), Uap1 (UDP-N-acetyl hexosamine pyrophosphorylase-1), Pcna (Proliferating Cell Nuclear Antigen), S100a11 (S100 calcium binding protein-A11); Ppp1 (Protein phosphatase1). Statistical significance was determined by one-sample *t*-test by comparing to the hypothetical value 1.0 (=no change). *p*-values are indicated as follows: * *p* < 0.05; ** *p* < 0.01; *** *p* < 0.001; **** *p* < 0.0001.

In most cases, the expressions of mRNA and protein were shown to go in parallel, though sometimes shifted in time (see Table 4 and Figure 5). We selected genes involved in intracellular trafficking (Eea1, Snx1) and import into cellular organelles (Kpna1, Tomm34), cellular junctions (Tjp2, Parva1), calcium homeostasis and signaling (Nucb1, Ip3r3, S100a11), biosynthesis of glycans (Nans, Uap1), DNA replication and repair (Pcna), cellular motility (Memo1), and phosphorylation (Ppp1), as well as a protein previously shown to be involved in vascular remodeling in response to altered oxygen conditions (Fhl1) [16]. Constant and intermittent hyperoxia were shown to have similar trends in mRNA expression in several cases, despite differences in absolute values, as seen for Eea1, Tomm34, Tjp2, Parva1, Nucb1, Aifm1, Nans, Uap1, Memo1, Fhl1, S100a11 and Pcna. Obvious differences were observed for Snx1 at 24 h, Kpna1 at 72 h, and Ppp1 at 72 h. Generally, it seems that a fast response (4 h) of lung endothelial cells to hyperoxia is an attempt to maintain homeostasis by counter-regulating a potential detrimental effect of high oxygen, as exemplified by a reduction in endocytosis (Eea1), a strengthened barrier by increasing tight junction proteins (Tjp2), an increased importance of calcium signaling and homeostasis (Nucb1, Ip3r3, S100a11), an increase in the biosynthesis of sialic acids (Nans, Uap1) (investigated in more detail by our group in a previous study [13]), and an attempt to improve the fidelity of DNA replication and DNA repair (Pcna). Many of these effects are reversed at later time points.

## 4. Discussion

The special properties of the pulmonary vascular beds allow adaptation to different requirements with regard to flow rates depending on cardiac output, thereby optimizing perfusion and gas exchange. The pulmonary endothelium regulates barrier function, vascular tone and immune responses, is involved in various signaling pathways, counteracts thrombosis, and has a special active metabolism [17]. Barrier function is maintained by multi-protein complexes, which form adherent junctions, tight junctions, and gap junctions, that control flow of fluids and transmigration of proteins and cells. Barrier disintegration leads to pulmonary edema, which is a hallmark of several lung diseases, including acute lung injury (ALI) and its worse form, acute respiratory distress syndrome (ARDS).

In accordance with being a syndrome, ARDS is triggered by different (direct and indirect) insults and appears in different phenotypes with various degrees of hypoxemia, endothelial and epithelial injury, inflammation and aberrant coagulation. Accordingly, there have been a lot of efforts to sub-characterize the condition with regard to sub-phenotypes and endo-types in order to provide better suitable therapies and prognosis [18]. Precision medicine approaches, for instance, have been helpful in distinguishing a hyperinflammatory versus an uninflamed endo-type characterized by different plasma levels of inflammatory biomarkers, such as IL-6, IL-8, sICAM-1, and sTNFRI, that exhibited different response to treatments and outcome [19].

Central to all ARDS treatment regimens is providing sufficient oxygenation. There has been considerable argumentation regarding whether this can only be achieved by very high oxygen saturation, and at the same time taking the risk of oxygen toxicity. A recent clinical trial compared liberal (target PaO_2_: 90–105 mm Hg; SpO_2_ >96%) and conservative (SpO_2_: 88–92%) oxygen therapy in ARDS patients, implying a possible worse outcome in the conservative-oxygenation strategy with regard to 90-days mortality [20]. However, responses to oxygen therapy can also be different in ARDS sub-phenotype groups depending on genetic factors and oxidative stress levels, which are linked to inflammation.

The pulmonary vasculature has the special capability of sensing oxygen. Hypoxic conditions lead to the vasoconstriction of small pulmonary arteries, while systemic arteries dilate (=hypoxic pulmonary vasoconstriction, HPV) [21]. This mechanism redirects blood flow to better ventilated areas. Hyperoxia, on the other hand, leads to vasoconstriction in systemic microcirculation and high concentrations of oxygen further induce toxicity in the lungs, which is an issue in patients ventilated with supraphysiological oxygen. Translational animal experiments have shown that high oxygen aggravates ventilation-induced lung injury (VILI) with regard to pulmonary edema and inflammation [6]. Baboons exposed to hyperoxia revealed the destruction of endothelial cells and alveolar type I cells, interstitial edema, and activation of neutrophils [22]. 

Despite a number of animal experiments using hyperoxia exposure followed by proteomic analysis [23,24,25], detailed studies of molecular effects of hyperoxia on isolated cell types have only been described for alveolar type II cells [26], but not for the pulmonary endothelium. 

In this study, we therefore aimed to decipher changes of the proteome in pulmonary endothelial cells in response to chronic constant and intermittent hyperoxia, which might help to better understand detrimental impact of oxygen on the organ lung.

Analysis of the mRNA levels of selected proteins revealed dynamic changes of expression starting from as early as 4 h until 72 h of exposure. For quantitative proteomic analysis, however, we chose exposure times of 24 h and 72 h. These data showed that constant and intermittent hyperoxia induce different responses with regard to the proteome. A total of 24 h of constant severe hyperoxia upregulated pathways related to RNA metabolism, cell cycle, mRNA splicing, cellular stress response, interleukin signaling, and apoptosis, and downregulated translation, processes involving small GTPases, TCA cycle and respiratory electron transport, VEGF (vascular endothelial growth factor) signaling, platelet activation, and cell junction organization. After 72 h of constant severe hyperoxia, enriched pathways shift to an upregulation of protein metabolism, ribosomes, and intracellular transport, while cell cycle, DNA repair, TCA cycle, respiratory electron transport, and cellular stress response are downregulated. These dynamics might reflect the situation encountered by a relatively fast start-up response of mRNAs (as measured at 4 h by qRT-PCR), that in many cases is counter-regulated at later time points and is also translated into proteins with the necessary time delay. Here, feedback mechanisms might play an important role, as can be anticipated as an example of “cell cycle”, which is upregulated at 24 h and again downregulated at 72 h 95% O_2_.

24 h of intermittent hyperoxia (0–95% O_2_) downregulates calcium homeostasis, responses to oxygen compounds, phosphorylation, and platelet activation. After 72 h (0–95% O_2_), posttranslational modifications such as neddylation and endocytosis are upregulated, while cell cycle, DNA repair, and p53 signaling are downregulated. Neddylation is a post-translational conjugation of the ubiquitin-like molecule neural precursor cell-expressed developmentally downregulated protein 8 (Nedd8) to different substrates, such as cullins, Akt, Hdac2, Hif1α, Hif2α, IKKγ, Traf6, Myd88, PPARγ, and Pcna, and affects transcription factors such as Nrf2 and NF-ΚB, the expression of pro-inflammatory cytokines, and barrier function [27].

It can be observed that under both conditions, long-term constant and intermittent hyperoxia, cells cease to proliferate, which is seen in a reduced cell count [28].

From what is already known, both conditions—constant and intermittent hyperoxia—will induce oxidative stress in the pulmonary endothelium, but the mechanisms and sources of ROS might be different. Molecular mechanisms of intermittent hyperoxia have not been elucidated in detail so far. In some of our previous studies, our group found that intermittent hyperoxia blunts the inflammatory response elicited by constant hyperoxia [29], activates the renin-angiotensin-system (RAS), and generates large amounts of peroxynitrite in the pulmonary endothelium [30]. Interestingly, nitric oxide (NO) has been shown to inhibit the activation of NF-ΚB induced by hyperoxia in neonatal pulmonary microvascular endothelial cells [31]. There is a mechanistic concept for molecular events in intermittent hypoxia proposed by Nanduri [32], according to which NADPH oxidase-derived ROS activates PLCγ and produces a calcium signal via inositol-3-phosphate, that ultimately activates HIF-1α. Interestingly, our proteomic study also highlights the inositol-3-phosphate receptor as a protein affected by hypoxic/hyperoxic O_2_ oscillations, implicating the role of calcium signaling under these conditions, and KEGG pathway enrichment analysis implicates HIF-1 pathway involvement at 24 h 0–95% O_2_. Further detailed mechanistic studies into these issues are certainly required.

Previous studies have shown that alternating oxygen between hypoxia and hyperoxia leads to the activation of signaling pathways that resemble hypoxic responses (such as HIF1 activation) without their detrimental side effects (“hyperoxic-hypoxic paradox”) [33]. Repeated oscillations between hyperoxia and hypoxia are believed to increase the ROS scavenger/ROS ratio, thereby protecting cells from ROS damage. Moreover, the oxygen oscillations present a therapeutical stress on mitochondria, encouraging the elimination of damaged organelles and the biogenesis of new, healthy mitochondria. The hypoxic periods allow the activation of HIF1, VEGF (angiogenesis, arteriogenesis), and stem cell proliferation. Clinical therapies making use of this phenomenon include Intermittent Hypoxic-Hyperoxic Treatment (IHHT) [34] and Hyperbaric Oxygen Treatment (HBOT) [35]. In a clinical setting, IHHT has been shown to improve the lipid profile and anti-inflammatory status in patients with metabolic syndrome [36]. An interesting issue for further mechanistic investigations might be the crosstalk between HIF and NF-ΚB transcription factors, which have been shown to be interlinked in a cell-type specific way [37].

In context of such mechanistic insights, an old therapeutical concept obtrudes, suggesting the use of pharmaceuticals or nutraceuticals to tackle oxidative stress and inflammation. There are many “natural” candidates, such as antioxidants resveratrol, N-acetyl cysteine (NAC) or anti-inflammatory sulforaphane from broccoli, curcuminoids or short-chain fatty acids (SCFA) acetate, propionate and butyrate, which are metabolites of a healthy gut microbiome. In addition, there is a large number of pharmaceutical drugs available. Interestingly, despite some promising results from preclinical in vitro and animal studies, the translation into human medicine is frequently difficult [38]. Resveratrol has been shown to induce apoptosis and autophagy in cancer cells [39] and attenuates inflammation in allergic asthma [40]. Similarly, SCFAs have been shown to exhibit anti-cancer and anti-inflammatory activity in cell cultures [41]. There is plenty of evidence that gut dysbiosis underlies many diseases, including diabetes mellitus, atherosclerosis, depression, and pulmonary arterial hypertension [42]. Curcuminoids are known to inactivate NF-KB and thereby decrease the transcription of pro-inflammatory mediators. In addition, they are capable of modulating the immune response and are promising drug candidates in cancer therapy [43]. Due to the chemical properties of these compounds, they are not readily bioavailable and are poorly assimilated upon ingestion. These problems with efficacy are addressed by efforts to encapsulate the substances and to use to lipid carriers for delivery to targets [44].

On the other hand, we are exposed to environmental toxins which have an impact on the antioxidant and inflammatory status of the respiratory system, thereby most likely also increasing the probability of developing ARDS during lung disease. One example are endocrine disruptors, which are part of plastics (“microplastics”, for example Bisphenol A) or are used as fungicides in agriculture (for example, Vinclozolin). These substances not only have an impact on the reproductive system, but also other organs including the lung by affecting Nrf2/NF-ΚB pathways [45,46].

Oxygen therapy in cancer patients seems to be a double-edged sword, and its benefits and potential harmful effects are a matter of continuous debate. Tumor microenvironments are frequently hypoxic due to rapid cell growth, and, despite the neovascularization, due to limited oxygen supply. Switching the metabolism of cancer cells to hypoxia plays a role in metastasis [47]. Hypoxia also fosters resistance to cytotoxic CD8+ T-cell (CTL)-attack by different mechanisms, and upregulates the expression of PD-L1 to increase CTL apoptosis. Immune checkpoint inhibitors (ICIs) have been developed that block PD-L1, PD-1, or CTLA-4. These drugs have been shown to improve overall survival in cancer patients, but have also adverse (cardiac) side effects [48]. Mechanisms of action are an increase in NLRP3, MyD88, and interleukin signaling inducing a cytokine storm. In order to reduce tumor hypoxia and also to increase ROS that might support cancer therapy, the application of oxygen has been proposed as a supplemental measure [49]. Overall, several studies have resulted in mixed outcomes; therefore, a general recommendation has not been given. A reason for this might be the challenge of fully understanding the regulation of oxygen homeostasis in the organism in its complexity, ROS signaling function, and detrimental side effects and the network of differentially regulated antioxidative response genes. Additional oxygen not only alleviates hypoxia, but can also induce inflammation; therefore, the right dosage reaching the target is difficult to obtain. Moreover, respiratory oxygen application efforts are made to enclose oxygen in microcapsules, which was shown to improve immune checkpoint blockades in pre-clinical studies [50]. This might also be an issue, especially in the context of anti-cancer drugs such as doxorubicin and angiogenesis inhibitors, where inflammation and endotheliotoxicity are significant risks [51].

### Limitations of the Study

This study was performed using murine primary pulmonary endothelial cells, which—as discussed previously—are prone to relatively rapid dedifferentiation in vitro after isolation from the lungs. We tried to tackle this problem by repurifying cells in cultures prior to the experiment, using endothelial-specific surface markers in order to make sure the cells maintained the endothelial properties. We always quantified changes in protein expression related to the normoxic condition at the same time point in order to eliminate changes in protein expression attributed to in vitro culturing.

It could be argued that another issue is that our “normoxic” condition (=21% O_2_) is not “physoxia” for lung endothelial cells (5–10% O_2_), but rather already a slightly hyperoxic condition. The pulmonary endothelium and other microvascular endothelial cells sense oxygen and its metabolism in an organ-specific way [34]. Reiterer et al. [34] show that maintaining cells at supra-physiological O_2_ levels impairs a normal response to hypoxia. However, in our study we merely investigate responses to severe hyperoxia and oscillations around a hyperoxic mean value compared to ambient O_2_ concentrations (21% O_2_).

A technical limitation of this study is owed to the fact that the yield of endothelial cells from mouse lung is low. For the sake of reducing animal numbers in the sense of the “3R Principle (Replacement-Reduction-Refinement of animal experiments”, we limited our proteomic analysis to the minimum of necessary replicates for a (semi-)quantitative assessment of changes in protein expression. However, results have been supported by independent mRNA expression analysis and are in good accordance with previous findings, as discussed in this paper.

## 5. Conclusions

Proteomic analysis of pulmonary endothelial cells reveals that exposure to constant and intermittent hyperoxia have different and time-dependent impacts on molecular events. Many processes seem to be blunted by the hypoxic/hyperoxic oscillation, which can be deduced from mRNA quantification and also from a much shorter list of proteins with significant changes of expression. However, there also seem to be differences in signaling pathways, which require further mechanistic studies into detailed molecular pathways.

## Figures and Tables

**Table 1:** Differentially expressed proteins (DEPs) after 24 h and 72 h exposure to constant severe hyperoxia. Murine lung endothelial cells were exposed for 24 h (**A**) and 72 h (**B**) to 95% O_2_. Semi-quantitative proteomic analysis revealed statistically significant changes in protein expression (blue: downregulated DEPs; red: upregulated DEPs). Results are log(2) fold changes compared to exposure under 21% O_2_ for the same period of time. Statistical evaluation: two-sample *t*-test assuming unequal variances.

(A) Hyperoxia 95% O_2_ vs. Normoxia 24 h						
Acc #	Gene Name	*t*-Test *p*-Value	log(2) [Fold Change]	Protein MW	Species	Protein Name
P51410	Rpl9	0.045581	−1.461	21,881.6	MOUSE	60S ribosomal protein L9
Q91YT0	Ndufv1	0.02777	−1.369	50,834.7	MOUSE	NADH dehydrogenase [ubiquinone] flavoprotein 1, mitochondrial
Q9R0Y5	Ak1	0.00099	−1.269	21,539.8	MOUSE	Adenylate kinase isoenzyme 1
P62911	Rpl32	0.03549	−1.164	15,859.9	MOUSE	60S ribosomal protein L32
Q545Q2	Surf4	0.00021	−1.156	30,381.3	MOUSE	Surfeit gene 4, isoform CRA_a
Q6ZQ38	Cand1	0.01071	−1.143	136,332.9	MOUSE	Cullin-associated NEDD8-dissociated protein 1
P22777	Serpine1	0.04550	−1.122	45,170.6	MOUSE	Plasminogen activator inhibitor 1
P60766	Cdc42	0.00012	−1.116	21,258.8	MOUSE	Cell division control protein 42 homolog
E9PUD2	Dnm1l	0.00539	−1.089	79,533.3	MOUSE	Dynamin-1-like protein
Q04447	Ckb	0.04549	−1.005	42,713.6	MOUSE	Creatine kinase B-type
Q3V3R1	Mthfd1l	0.02608	1.012	105,729.7	MOUSE	Monofunctional C1-tetrahydrofolate synthase, mitochondrial
B2RQC6	Cad	0.04433	1.012	243,240.6	MOUSE	CAD protein
Q3ULB1	Tes	0.02179	1.022	46,587.2	MOUSE	Testin
O35350	Capn1	0.03432	1.025	82,107	MOUSE	Calpain-1 catalytic subunit
Q3U417	Ppfibp1	0.00443	1.045	108,539.3	MOUSE	PTPRF interacting protein, binding protein 1 (liprin beta 1)
P24270	Cat	0.03979	1.082	59,795.8	MOUSE	Catalase
Q3TJ95	Cltb	0.04187	1.095	23,116.5	MOUSE	Clathrin light chain
Q9D6Z1	Nop56	0.01453	1.107	64,464.9	MOUSE	Nucleolar protein 56
Q8BJ71	Nup93	0.03429	1.126	93,281.9	MOUSE	Nuclear pore complex protein Nup93
P55302	Lrpap1	0.01909	1.152	42,215.4	MOUSE	Alpha-2-macroglobulin receptor-associated protein
Q9D2R0	Aacs	0.00194	1.161	75,200.9	MOUSE	Acetoacetyl-CoA synthetase
Q8BXZ1	Tmx3	0.03757	1.163	51,848.5	MOUSE	Protein disulfide-isomerase TMX3
Q9ER72	Cars1	0.02970	1.178	94,860.6	MOUSE	Cysteine—tRNA ligase, cytoplasmic
Q3T9V8	Dctn1	0.00045	1.184	136,879.4	MOUSE	Dynactin subunit 1
P97742	Cpt1a	0.01288	1.191	88,252.3	MOUSE	Carnitine O-palmitoyltransferase 1, liver isoform
Q91XH5	Spr	0.03473	1.220	27,928.4	MOUSE	Sepiapterin reductase
Q921X9	Pdia5	0.02308	1.273	59,267.7	MOUSE	Protein disulfide-isomerase A5
B8X349	Spag9	0.04057	1.307	146,132.7	MOUSE	JNK-interacting leucine zipper protein long form
Q9Z0X1	Aifm1	0.00500	1.344	66,766.1	MOUSE	Apoptosis-inducing factor 1, mitochondrial
Q6ZQ84	mKIAA0617	0.02823	1.385	91,277.7	MOUSE	MKIAA0617 protein (Fragment)
Q3UW53	Niban1	0.03476	1.399	102,649.9	MOUSE	Protein Niban
Q8K1N2	Phldb2	0.03488	1.401	141,486.8	MOUSE	Pleckstrin homology-like domain family B member 2
O35226	Psmd4	0.00918	1.407	40,704.1	MOUSE	26S proteasome non-ATPase regulatory subunit 4
O70318	Epb41l2	0.00888	1.589	109,940.4	MOUSE	Band 4.1-like protein 2
Q02819	Nucb1	0.01474	1.640	53,409.1	MOUSE	Nucleobindin-1
Q91X52	Dcxr	0.00908	1.673	25,746.1	MOUSE	L-xylulose reductase
Q3TRX4	Palm	0.00689	1.684	36,750.1	MOUSE	Isoform 2 of Paralemmin-1
P58871	Tnks1bp1	0.00854	1.688	181,826.4	MOUSE	182 kDa tankyrase-1-binding protein
O08759	Ube3a	0.00112	1.717	99,820.4	MOUSE	Ubiquitin-protein ligase E3A
Q62376	Snrnp70	0.01119	1.728	51,992.5	MOUSE	U1 small nuclear ribonucleoprotein 70 kDa
Q9EP71	Rai14	0.00394	1.753	108,853.2	MOUSE	Ankycorbin
Q3UF75	Parva	0.00386	1.825	38,361.5	MOUSE	Alpha-parvin
Q3UDJ2	Sgpl1	0.00440	1.902	63,649.8	MOUSE	Sphingosine-1-phosphate lyase 1
Q78IK4	Apool	0.00092	2.050	29,261	MOUSE	MICOS complex subunit Mic27
Q9ERU9	Ranbp2	0.00396	2.196	341,123.8	MOUSE	E3 SUMO-protein ligase RanBP2
B2RWW2	Golgb1	0.00383	2.265	370,113.8	MOUSE	Golgb1 protein
Q8BL66	Eea1	0.00080	2.357	160,915.9	MOUSE	Early endosome antigen 1
Q9CYG7	Tomm34	0.00028	2.407	34,278.3	MOUSE	Mitochondrial import receptor subunit TOM34
Q3U962	Col5a2	0.02207	2.539	145,019.6	MOUSE	Collagen alpha-2(V) chain
Q922J3	Clip1	0.03234	2.757	155,815.2	MOUSE	CAP-Gly domain-containing linker protein 1
Q3THU7	Clta	0.04798	2.766	23,563.9	MOUSE	Clathrin light chain
Q9Z0U1	Tjp2	0.00852	2.854	131,281	MOUSE	Tight junction protein ZO-2
**(B) Hyperoxia 95% O_2_ vs. Normoxia 72 h**						
**Acc #**	**Gene Name**	***t*** **-Test** ***p*-Value**	**log(2)** **[Fold Change]**	**Protein MW**	**Species**	**Protein Name**
P52293	Kpna2	0.00578	−3.516	57,928.6	MOUSE	Importin subunit alpha-1
Q00915	Rbp1	0.01992	−2.516	15,846.3	MOUSE	Retinol-binding protein 1
P50543	S100a11	0.00185	−1.587	11,082.8	MOUSE	Protein S100-A11
P17918	Pcna	0.00288	−1.448	28,785.1	MOUSE	Proliferating cell nuclear antigen
O35682	Myadm	0.00299	−1.363	35,284.9	MOUSE	Myeloid-associated differentiation marker
Q3UJR8	Btf3	0.01726	−1.295	17,699.2	MOUSE	Transcription factor BTF3
P46061	Rangap1	0.03792	−1.243	63,531.5	MOUSE	Ran GTPase-activating protein 1
Q8K2B3	Sdha	0.00396	−1.237	72,586.1	MOUSE	Succinate dehydrogenase [ubiquinone] flavoprotein subunit, mitoch.
Q4KML4	Abracl	0.00194	−1.149	9030.5	MOUSE	Costars family protein ABRACL
Q3TA69	Rap1gds1	0.01416	−1.124	66,076.1	MOUSE	Rap1 GTPase-GDP dissociation stimulator 1
Q04447	Ckb	0.01411	−1.103	42,713.6	MOUSE	Creatine kinase B-type
Q8R2Y2	Mcam	0.00035	−1.095	71,546.1	MOUSE	Cell surface glycoprotein MUC18
Q564E8	Rpl4	0.02582	−1.091	47,154.1	MOUSE	Ribosomal protein L4
P30999	Ctnnd1	0.00303	−1.050	104,925.7	MOUSE	Catenin delta-1
P83940	Eloc	0.04477	−1.017	12,473.3	MOUSE	Elongin-C
Q99LJ0	Cttnbp2nl	0.02629	1.005	69,841.7	MOUSE	CTTNBP2 N-terminal-like protein
Q64521	Gpd2	0.00564	1.009	80,954.5	MOUSE	Glycerol-3-phosphate dehydrogenase, mitochondrial
P31230	Aimp1	0.00542	1.036	33,997.6	MOUSE	Aminoacyl tRNA synthase complex-interacting multifunctional protein 1
Q05816	Fabp5	0.00502	1.064	15,137.6	MOUSE	Fatty acid-binding protein, epidermal
P97314	Csrp2	0.02217	1.075	20,926	MOUSE	Cysteine and glycine-rich protein 2
Q3TW96	Uap1l1	0.02466	1.130	56,614.3	MOUSE	UDP-N-acetylhexosamine pyrophosphorylase-like protein 1
Q78IK4	Apool	0.03345	1.162	29,261	MOUSE	MICOS complex subunit Mic27
O88543	Cops3	0.03558	1.174	47,832.5	MOUSE	COP9 signalosome complex subunit 3
Q6PHZ2	Camk2d	0.01028	1.236	56,369.9	MOUSE	Calcium/calmodulin-dependent protein kinase type II subunit delta
P97447	Fhl1	0.00893	1.265	31,889.1	MOUSE	Four and a half LIM domains protein 1
Q3U1W3	Adam9	0.00134	1.308	91,848	MOUSE	Disintegrin and metalloproteinase domain-containing protein 9
Q9DBL1	Acadsb	0.01594	1.349	47,874.5	MOUSE	Short/branched chain specific acyl-CoA dehydrogenase, mitochondrial
Q03145	Epha2	0.01586	1.402	108,853.2	MOUSE	Ephrin type-A receptor 2
O70439	Stx7	0.02471	1.602	29,821	MOUSE	Syntaxin-7
Q9ER00	Stx12	0.00453	1.614	31,195.5	MOUSE	Syntaxin-12
Q3UDJ2	Sgpl1	0.04139	1.631	63,649.8	MOUSE	Sphingosine-1-phosphate lyase 1
Q5SUH6	Clint1	0.02425	1.736	69,759.8	MOUSE	Clathrin interactor 1
O35382	Exoc4	0.03902	1.745	110,545.9	MOUSE	Exocyst complex component 4
Q8BL66	Eea1	0.01546	1.863	160,915.9	MOUSE	Early endosome antigen 1
P41105	Rpl28	0.00425	2.118	15,733.6	MOUSE	60S ribosomal protein L28
Q6PB44	Ptpn23	0.00004	2.754	185,218	MOUSE	Tyrosine-protein phosphatase non-receptor type 23

**Table 2:** Differentially expressed proteins (DEPs) after 24 h and 72 h exposure to hypoxic-hyperoxic oscillations. Murine lung endothelial cells were exposed for 24 h (**A**) and 72 h (**B**) to 0–95% O_2_ oscillations. Semi-quantitative proteomic analysis revealed statistically significant changes in protein expression (blue: downregulated DEPs; red: upregulated DEPs). Results are log(2) fold changes compared to exposure under 21% O_2_ for the same period of time. Statistical evaluation: two-sample *t*-test assuming unequal variances.

(A) Oscillation 0–95% O_2_ vs. Normoxia 24 h						
Acc #	Gene Name	*t*-Test *p*-Value	log(2) [Fold Change]	Protein MW	Species	Protein Name
Q91YT0	Ndufv1	0.01774	−2.093	50,834.7	MOUSE	NADH dehydrogenase [ubiquinone] flavoprotein 1, mitochondrial
Q9DBR7	Ppp1r12a	0.02466	−2.034	114,997.2	MOUSE	Protein phosphatase 1 regulatory subunit 12A
Q53WR6	Glg1	0.01798	−1.668	133,735.2	MOUSE	Golgi apparatus protein 1
Q99KP6	Prpf19	0.01909	−1.634	55,239.3	MOUSE	Pre-mRNA-processing factor 19
P97447	Fhl1	0.00851	−1.613	31,889.1	MOUSE	Four and a half LIM domains protein 1
Q3TZU7	Snx9	0.04307	−1.594	66,516.2	MOUSE	Sorting nexin
P70227	Itpr3	0.03133	−1.570	304,277.6	MOUSE	Inositol 1,4,5-trisphosphate receptor type 3
Q9DBF1	Aldh7a1	0.01686	−1.352	58,862	MOUSE	Alpha-aminoadipic semialdehyde dehydrogenase
P47856	Gfpt1	0.02266	−1.334	78,539.8	MOUSE	Glutamine–fructose-6-phosphate aminotransferase [isomerizing] 1
Q99J77	Nans	0.00815	−1.271	40,024.5	MOUSE	Sialic acid synthase
Q9CQC6	Bzw1	0.02597	−1.238	48,043.6	MOUSE	eIF5-mimic protein 2
P29268	Ccn2	0.00962	−1.010	37,824.6	MOUSE	CCN family member 2
Q6PAC1	Gsn	0.04512	1.059	80,763.2	MOUSE	Gelsolin
Q543N3	Lasp1	0.04676	1.073	29,994.7	MOUSE	LIM and SH3 domain protein 1
**(B) Oscillation 0–95% O_2_ vs. Normoxia 72 h**						
**Acc #**	**Gene Name**	***t*-Test** ***p*-Value**	**log(2)** **[Fold Change]**	**Protein MW**	**Species**	**Protein Name**
Q91VH6	Memo1	0.00606	−2.074	33,692.4	MOUSE	Protein MEMO1
P17918	Pcna	0.00344	−1.579	28,785.1	MOUSE	Proliferating cell nuclear antigen
O88544	Cops4	0.01743	−1.564	46,285.2	MOUSE	COP9 signalosome complex subunit 4
P52293	Kpna2	0.01117	−1.272	57,928.6	MOUSE	Importin subunit alpha-1
P46638	Rab11b	0.00025	1.014	24,489.7	MOUSE	Ras-related protein Rab-11B
Q6P6L0	Filip1l	0.00871	1.097	129,773.6	MOUSE	Filamin A-interacting protein 1-like
Q3TW96	Uap1l1	0.01494	1.189	56,614.3	MOUSE	UDP-N-acetylhexosamine pyrophosphorylase-like protein 1
Q3MIA8	Gps1	0.01047	1.347	55,163.7	MOUSE	COP9 signalosome complex subunit 1
Q5SWZ5	Mprip	0.00666	1.353	257,288.4	MOUSE	Myosin phosphatase Rho-interacting protein

**Table 3:** Differentially expressed proteins (DEPs) after 24 h and 72 h exposure to normoxia (21% O_2_) compared to baseline (0 h). Murine lung endothelial cells were exposed for 24 h (**A**) and 72 h (**B**) to 21% O_2_. Semi-quantitative proteomic analysis revealed statistically significant changes in protein expression (blue: downregulated DEPs; red: upregulated DEPs). Results are log(2) fold changes compared to baseline (=start of experiment, Time = 0). Statistical evaluation: two-sample *t*-test assuming unequal variances.

(A) Baseline vs. Normoxia 24 h						
Acc #	Gene Name	*t*-Test *p*-Value	log(2) [Fold Change]	Protein MW	Species	Protein Name
E9QA15	Cald1	0.044003	−1.097	89,274.1	MOUSE	Protein Cald1
P22777	Serpine1	0.042341	1.145	45,170.6	MOUSE	Plasminogen activator inhibitor 1
Q62219	Tgfb1i1	0.004814	2.049	50,101.1	MOUSE	Transforming growth factor beta-1-induced transcript 1 protein
P55284	Cdh5	0.03317	1.556	87,903.7	MOUSE	Cadherin-5
P47856	Gfpt1	0.023897	1.678	78,539.8	MOUSE	Glutamine–fructose-6-phosphate aminotransferase [isomerizing] 1
**(B) Baseline vs. Normoxia 72 h**						
**Acc #**	**Gene Name**	***t*-Test** ***p*-Value**	**log(2)** **[Fold Change]**	**Protein MW**	**Species**	**Protein Name**
P29533	Vcam1	0.04293	−1.191	81,318.2	MOUSE	Vascular cell adhesion protein 1
P28660	Nckap1	0.02359	1.019	128,785.1	MOUSE	Nck-associated protein 1
P52293	Kpna2	0.01242	1.092	57,928.6	MOUSE	Importin subunit alpha-1
P31938	Map2k1	0.01551	1.111	43,474.4	MOUSE	Dual specificity mitogen-activated protein kinase kinase 1
P50543	S100a11	0.02928	1.124	11,082.8	MOUSE	Protein S100-A11
Q8BKC5	Ipo5	0.04751	1.146	123,592.2	MOUSE	Importin-5
Q8R016	Blmh	0.02996	1.164	52,511.6	MOUSE	Bleomycin hydrolase
P30999	Ctnnd1	0.02446	1.266	104,925.7	MOUSE	Catenin delta-1
Q62470	Itga3	0.00063	1.292	116,746.2	MOUSE	Integrin alpha-3
D3YVF0	Akap5	0.00832	1.304	79,397.3	MOUSE	A-kinase anchor protein 5
Q8R3B1	Plcd1	0.02847	1.308	85,873.9	MOUSE	1-phosphatidylinositol 4,5-bisphosphate phosphodiesterase delta-1
Q91VH6	Memo1	0.02678	1.309	33,692.4	MOUSE	Protein MEMO1
Q03145	Epha2	0.00533	1.365	108,853.2	MOUSE	Ephrin type-A receptor 2
Q8R2Y2	Mcam	0.00016	1.573	71,546.1	MOUSE	Cell surface glycoprotein MUC18
O35345	Kpna6	0.02067	1.634	59,964.9	MOUSE	Importin subunit alpha-7
E9QNA7	Sorbs1	0.04557	1.705	82,875.8	MOUSE	Sorbin and SH3 domain-containing protein 1
Q62219	Tgfb1i1	0.00452	1.715	50,101.1	MOUSE	Transforming growth factor beta-1-induced transcript 1 protein
Q8CGB9	Ide	0.04366	2.118	117,696	MOUSE	Insulin degrading enzyme
P37889	Fbln2	0.03797	2.344	131,834.9	MOUSE	Fibulin-2
P51655	Gpc4	0.01642	2.629	62,586.8	MOUSE	Glypican-4

**Table 4:** Proteins identified as DEPs in proteomic analysis and selected for further quantification of mRNA expression at time points 4 h, 24 h and 72 h. Significant upregulation and downregulation of DEPs as determined by proteomic analysis (protein), and qRT-PCR (mRNA) is indicated by arrows.

Protein	Location	Function and Related Pathways *	mRNA		Protein	
			95% O_2_	0–95% O_2_	95% O_2_	0–95% O_2_
*Eea1* *Early endosome antigen*	early endosome, cytosol	endosomal trafficking, endocytosis, vesicle fusion	4 h ↓ 24 h ↑ 72 h ↑		24 h ↑ 72 h ↑	
*Snx1* *Sorting nexin*	endosomes	intracellular trafficking, protein recycling to plasma membrane, retrograde transport from endosomes to the Trans-Golgi-Network (TGN)	24 h ↑	24 h ↓		24 h ↓
*Kpna1* *Importin-1*	nuclear pores	nuclear protein import	4 h ↑ 24 h ↑ 72 h ↑			72 h ↓
*Tomm34* *Translocase of outer mitochondrial membrane*	outer mitochondrial membrane, cytosol	import of preproteins into mitochondria	24 h ↑ 72 h ↑		24 h ↑	
*Zo2 (Tjp2)* *Tight junction protein 2*	tight junctions adherens junctions	cell adhesion, apoptotic cleavage of cellular proteins	4 h ↑	4 h ↑	24 h ↑	
*Parva* *Parvin-*	focal adhesion nucleus, cytosol	enables actin binding activity, lamellipodium	4 h ↓ 24 h ↑	4 h ↓	24 h ↑	
*Nucb1* *nucleobindin*	Golgi apparatus	calcium-binding EF-hand protein family, Golgi calcium homeostasis, calcium-regulated signal transduction, non-receptor guanine nucleotide exchange factor (GEF), binds and activates G-proteins	24 h ↑		24 h ↑	
*Aifm1* *Apoptosis- inducing factor1 mitochondrion associated*	nucleus, cytosol, mitochondria	Triggers chromatin condensation and DNA fragmentation to induce programmed cell death; regulates permeability of the mitochondrial membrane; acts as NADH oxidase	4 h ↑ 24 h ↓ 72 h ↓	4 h ↑	24 h ↑	
*Fhl1* *Four and a half Lim domains*	cytosol, nucleus, plasma membrane	zink-finger domain protein, cell differentiation, establishment of localization	4 h ↓ 72 h ↓	4 h ↓ 24 h ↑ 72 h ↓	72 h ↑	24 h ↓
*Ip3r3* *Inositol 1,4,5 triphosphate receptor type 1*	endoplasmic reticulum (ER), nucleus, plasma membrane	mediates calcium release from ER following stimulation with inositol-1,4,5-triphosphate		24 h ↓	72 h ↑	24 h ↓
*Pcna* *Proliferating cell nuclear antigen*	nucleus, cytoskeleton	cofactor of DNA polymerase activity, in response to DNA damage the protein is ubiquitinated and involved in DNA repair, role in mitotic G1 phase and G1/S transition	4 h ↑ 24 h ↓ 72 h ↓	4 h ↑ 72 h ↓	72 h ↓	72 h ↓
*Ppp1ca* *Protein phosphatase 1 catalytic subunit alpha*	nucleus, cytosol, plasma membrane	serine/threonine specific phosphatase, broad functions including cell division, glycogen metabolism, protein synthesis	24 h ↑ 72 h ↑			
*Nans* *N-Acetylneuraminate synthase*	cytosol	biosynthetic pathways of sialic acids	24 h ↓	4 h ↑ 24 h ↓ 72 h ↓		24 h ↓
*Memo1* *Mediator of cell motility*	intracellular, plasma membrane, vesicles	control of cell migration by relaying chemotactic signals to the microtubule cytoskeleton	4 h ↑ 24 h ↑	4 h ↑ 24 h ↑ 72 h ↓		72 h ↓
*UAP1* *UDP-N-acetylhexosamine pyrophosphorylase-like protein 1*	plasma membrane, nucleus, cytosol	Involved in the biosynthesis of UDP-N-acetylglucosamine	4 h ↑ 72 h ↓	4 h ↑ 24 h ↓ 72 h ↑	72 h ↑	72 h ↑
*S100a11* *S100 calcium-binding protein A11-calgizzarin*	nucleus, cytoplasm	2 EF-hand binding motifs, cell cycle progression, differentiation, motility, tubulin polymerization	4 h ↑ 24 h ↑ 72 h ↓	4 h ↑	72 h ↓	

* Sources: MGI (mouse genome informatics) mouse genome database, Gene Cards human gene database (www.genecards.org (accessed on 11 July 2022), Uniprot Database (www.uniprot.org (accessed on 11 July 2022)).

## Data Availability

The data presented in this study are available in the article and Supplementary Materials.

## Supplementary Materials

The following supporting information can be downloaded at: https://www.mdpi.com/article/10.3390/antiox11122349/s1, Supplementary Data S1: qRT-PCR primers for expression analysis of mRNAs; Supplementary Data S2: enrichment analysis using GO, KEGG and Reactome data bases of individual treatments at different time points.
