# Supplementary material for: Effects of Hyperoxia and Hyperoxic Oscillations on the Proteome of Murine Lung Microvascular Endothelium
Source: Antioxidants (Basel). 2022 Nov 28;11(12):2349. doi: 10.3390/antiox11122349 (PMC9774699; doi:10.3390/antiox11122349)
Supplement: Supplementary file 1 [file antioxidants-11-02349-s001.zip › Supplementary Data S1.pdf]

| | | |
|---|---|---|
| Eea1 | fw: | 5'-AAACCAGCTAAGGAGTGAACTTG-3' |
| | rv: | 5'-GTGGGTGTAGTCTAGGTCTTTCT-3' |
| Uap1 | fw: | 5'-ACTCCCAGGGGCACTTCATTA-3' |
| | rv: | 5'-GGCCTCTGACTTTCCATTTGT-3' |
| Kpna1 | fw: | 5'- TGGAGTTCCTCAAACGAAAAGAA-3' |
| | rv: | 5'- TTTGTCAGGACCCAAGCTGAT-3' |
| NANS | fw: | 5'-CACCAAGGAGACATAGATGTGGC-3' |
| | rv: | 5'-CTCTTCTGAAACTTAGCGCAGT-3' |
| Memo1 | fw: | 5'-GGATACACATACTGTGGGTCCT-3' |
| | rv: | 5'-CAGGGGCACATGATGGGAAG-3' |
| IP3R3 | fw: | 5'-CGAGATTGCCTCTTCAAAGTGT-3' |
| | rv: | 5'-GTCCTGTTTAGTCTGCTTGGC-3' |
| Snx1 | fw: | 5'-ACAGTGGAACTATCCCTGGAC-3' |
| | rv: | 5'-TTCTCAGGATCGGTGATACCAA-3' |
| Ppp1 | fw: | 5'-GACCAAGGTGAAGTTCGACGA-3' |
| | rv: | 5'-ACATTGGCGTAATTGATGTCGG-3' |
| Fhl1 | fw: | 5'-TAAGAATCGCTACTGGCACGA-3' |
| | rv: | 5'-AATGGCCTTGAAGCACCCTTT-3' |
| Tomm34 | fw: | 5'-ATGGCCCCCAAACTCTCAGA-3' |
| | rv: | 5'-CGGTTGGAGTACAGAACACTTT-3' |
| Tjp2 | fw: | 5'-ATGGGAGCAGTACACCGTGA-3' |
| | rv: | 5'-GCTGAACGGCAAACGAATGG-3' |
| Parva1 | fw: | 5'-TCCCCAAATCACCCACTCC-3' |
| | rv: | 5'-AGGTTGATGGCGTTCATTCCT-3' |
| Nucb1 | fw: | 5'-ACCACCTCTACTGATGCTGTC-3' |
| | rv: | 5'-CTAGCACGTTGATGACCTCCT-3' |
| Aifm1 | fw: | 5'-TCCAGAGGCCGAAACAGAG-3' |
| | rv: | 5'-CAGCTCCTATTGTTGATAAGCCC-3' |
| Pcna | fw: | 5'-TTTGAGGCACGCCTGATCC-3' |
| | rv: | 5'-GGAGACGTGAGACGAGTCCAT-3' |

| | | |
|---|---|---|
| S100a11 | fw: | 5‘-GCGGGAAGGATGGAAACAACA-3‘ |
| | rv: | 5‘-TCATCATGCGGTCAAGGACAC-3’ |
| Actb | fw: | 5’-AGCCATGTACGTAGCCATC-3’ |
| | rv: | 5’-CTCTCAGCTGTGGTGGTGA-3’ |
| Gapdh | fw: | AGGTCGGTGTGAACGGATTTG |
| | Rv: | TGTAGACCATGTAGTTGAGGTCA |
